# Genome-wide identification of *AAAP* gene family and expression analysis in response to saline-alkali stress in foxtail millet (*Setaria italica L.*)

**DOI:** 10.1038/s41598-024-53242-6

**Published:** 2024-02-07

**Authors:** Huimin Wang, Yun Li, Zhenqing Guo, Xiaoke Zhou, Yuxue Zhao, Yucui Han, Xiaohu Lin

**Affiliations:** 1 College of Agronomy and Biotechnology/Hebei Key Laboratory of Crop Stress Biology, Hebei Normal University of Science and Technology, Qinhuangdao, 066000 China; 2 Research Center of Rural Vitalization, Hebei Normal University of Science and Technology, Qinhuangdao, 066000 China

**Keywords:** Computational biology and bioinformatics, Genetics, Plant sciences

## Abstract

Amino acid/auxin permease (AAAP) genes encode a large family of protein transporters that play important roles in various aspects of plant growth and development. Here, we performed genome-wide identification of members in the foxtail millet (*Setaria italica L.*) AAAP family (SiAAAP) and their saline-alkali stress-induced expression patterns, resulting in the identification of 65 SiAAAP genes, which could be divided into eight subfamilies. Except for SiAAAP65, the remaining 64 genes were located on nine chromosomes of foxtail millet. Gene structure and conserved motif analyses indicated that the members in the same subfamily are highly conserved. Gene duplication event analysis suggested that tandem duplication may be the main factor driving the expansion of this gene family, and Ka/Ks analysis indicated that all the duplicated genes have undergone purifying selection. Transcriptome analysis showed differential expression of SiAAAPs in roots, stems, leaves, and tassel inflorescence. Analysis of cis-acting elements in the promoter indicated that SiAAAPs contain stress-responsive cis-acting elements. Under saline-alkali stress, qRT-PCR analysis showed that SiAAP3, SiLHT2, and SiAAP16 were differentially expressed between salt-alkali tolerant millet variety JK3 and salt-alkali sensitive millet variety B175. These results suggest that these genes may be involved in or regulate the response to saline-alkali stress, providing a theoretical basis for further studying the function of SiAAAPs.

## Introduction

Nitrogen is an essential mineral nutrient usually transported in plants in the form of amino acids. Changes in amino acid levels in plants affect many growth regulatory activities and are closely related to plant growth, development, and resistance to stress. Auxin is universally present in all plants as a necessary plant hormone for their survival. Amino acids and auxin need to be transported across cell membranes to exert their functions in different organs, which relies on specific carrier proteins on the plasma membrane, mainly amino acid transporter proteins (AATs)^1^. Currently, two families of AATs have been identified, namely the amino acid/auxin permease (*AAAP*) and amino acid polyamine choline gene family^2^. AAAP protein is an enzyme mediating the movement of a variety of amino acids and auxin into and out of cells^3^, and participates in regulating the transmembrane structure of amino acids and long-distance transport of amino acids in the body, as well as other life processes^4^.

Systematic analysis of the *AAAP* family can help understand the genes having undergone significant expansion or contraction in plants and the positive selection during evolution, so as to identify the genes related to the environmental adaptability of plants. Based on the similarities between sequences and the characteristics of conserved domains, the *AAAP* family can be divided into eight subfamilies, including aminoacid permeases (AAPs), lysine histidine transporters (LHTs), γ-aminobutyric acid transporters (GATs), proline transporters (ProTs), putative auxin transporters (AUXs), similar to ANT1-like aromatic and neutral amino acid transporters (ANTs), and amino acid transporter-like (ATLa, ATLb)^2,4,5^.Previous studies have shown that different subfamilies have low sequence similarities, but all *AAAP* genes share the same Aa_trans conserved domain (PF01490). Since the identification of the first *AAAP* gene in mammals, research on *AAAP* family genes has been gradually extended to plants, and has gradually developed from *Arabidopsis thaliana* to some important food crops, resulting in isolation, cloning, and functional analysis of more *AAAP* genes in various crops^6,7^. It has been demonstrated that *AAAP* genes directly or indirectly participate in regulating different developmental and physiological processes in plants^8,9^. Treatment with abscisic acid, salicylic acid, methyl jasmonate, sodium chloride or amino acids up-regulated the expression of *PgLHT* gene in Panax ginseng and significantly accelerated the growth rate of root hair^10^. Short-term salt stress greatly increased the expression level of *HvProT* in the roots of *Hordeum vulgare L.*^11^. *HvProT2* can affect the accumulation of intracellular glycinebetaine (GB) by regulating its transport in the plasma membrane of leaves and roots, thereby promoting plant tolerance to salt stress^12^. Overexpression of *GmProT1* and *GmProT2* in *Glycine max* (Gm) could significantly alleviate leaf damage caused by salt and drought stress^13^. The expression of *CsGAT* gene in *Camellia sinensis* (Cs) improved the conversion of glutamic acid (Glu) to gamma-aminobutyric acid (GABA) and enhanced the transport of GABA, thus maintaining high nitrogen availability^14^. These findings indicate the potential ability of *AAAP* gene family members to resist abiotic stresses.

As one of the main grain crops in northern China, foxtail millet (*Setaria italica L.*) is characterized by a small genome, short life cycle, self-pollination^15–17^, and strong resistance to drought and saline-alkali^18,19^. It is a typical environment-friendly crop and also a model plant for studying C4 cereal crops. In recent years, the whole genome sequencing of foxtail millet has been completed and published^17,20^, making it possible to excavate important gene functions in foxtail millet and study its stress response and molecular regulation mechanism. So far, there have been no reports on studying the *AAAP* gene family in millet crops. Here, we performed a comprehensive bioinformatics analysis of the *AAAP* family in foxtail millet, including the identification of gene family members, gene chromosome location, gene structure, phylogeny, gene GO and KEGG enrichment analysis, gene expression patterns in different tissues and under saline-alkali stress. The results provide a basis for further gene function study and genetic improvement of foxtail millet.

## Results

### Identification of *SiAAAP* family

Blast was conducted to compare the AAAP protein sequences in *Arabidopsis* with the protein sequences of foxtail millet, and then the conserved domain of foxtail millet AAAP protein sequences was identified. Finally, 65 high-confidence non-redundant *AAAP* genes (*SiAAAPs*) were obtained. These genes were renamed based on their chromosomal positions. The physicochemical characteristics of SiAAAPs (Table 1) show that their amino acid sequence length was similar to the molecular weight of protein. For example, *SiAAAP19* has the smallest amino acid sequence (255 aa) and *SiAAAP63* has the largest amino acid sequence (584 aa), which corresponded to the molecular weight of 27237.34 kDa and 62588.7 kDa, respectively. Moreover, SiAAAPs exhibited extensive variations in isoelectric point and instability index. The isoelectric point ranged from 4.94 (*SiAAAP7*) to 9.99 (*SiAAAP10*), among which 10 members had isoelectric point < 7 (acidic), and the remaining 55 members had isoelectric point > 7 (alkaline). These results indicated the presence of abundant basic proteins in SiAAAPs. The stability index ranged from 27.55 (*SiAAAP25*) to 57.36 (SiAAAP21), and 78% of the members had stability index < 40, indicating that they are relatively stable. The aliphatic index ranged from 54.18 (*SiAAAP22*) to 127.66 (*SiAAAP2*). A higher aliphatic index value represents higher thermal stability of AAAP proteins. In terms of grand average of hydropathicity (GRAVY), all SiAAAPs showed GRAVY ranging from 0.158 (*SiAAAP21*) to 0.886 (*SiAAAP4*), indicating that they are hydrophobic proteins. Subcellular localization results demonstrated that 62 of the 65 SiAAAPs are located in the plasma membrane and the remaining three SiAAAPs are located in the vacuole membrane. Prediction results of signal peptide and transmembrane region showed absence of signal peptides in *AAAP* family, but there were transmembrane regions. SiAAAPs usually had 9–11 transmembrane domains (TMs), but some members had quite different TMs, such as *SiAAAP63* (5), *SiAAAP48* (7), *SiAAAP56* (7), *SiAAAP7* (8), *SiAAAP21* (8), *SiAAAP23* (8), and *SiAAAP51* (8). These results suggested that these family members may be non-secretory proteins that cannot guide protein transmembrane transport, but can perform membrane protein functions in intracellular and extracellular signal transduction, and a decrease in the number of TMs indicates possible variations in their functions.

**Table 1:** Information of 65 *SiAAAP* genes in foxtail millet.

Sequence ID	Gene name	Number of amino acid	Molecular weight	Theoretical pI	Instability index	Aliphatic index	Grand average of hydropathicity	Subcellular localization	Signal peptide	Transmembrane region
KQL27687	*SiAAAP1*	565	61,339.92	7.05	52.35	103.91	0.263	plas	–	9
KQL28349	*SiAAAP2*	458	49,715.48	6.19	29.85	127.66	0.777	plas	–	10
KQL28894	*SiAAAP3*	456	50,249.23	9.23	34.78	97.98	0.506	plas	–	10
KQL30891	*SiAAAP4*	425	44,847.08	6.74	30.79	116.33	0.886	plas	–	11
KQL31276	*SiAAAP5*	463	49,713.66	7.97	31.73	95.59	0.539	plas	–	10
KQL31315	*SiAAAP6*	455	48,693.09	8.11	33.39	113.14	0.708	plas	–	11
KQL31857	*SiAAAP7*	548	59,626.48	4.94	40.17	101.39	0.293	plas	–	8
KQL31859	*SiAAAP8*	518	55,405.32	5.48	33.31	109	0.551	plas	–	11
KQL22378	*SiAAAP9*	483	52,720.31	8.85	29.83	99.96	0.452	plas	–	9
KQL23031	*SiAAAP10*	498	52,366.62	9.99	55.59	111.33	0.64	plas	–	11
KQL23069	*SiAAAP11*	349	38,178.87	6.3	46.54	113.98	0.753	plas	–	9
KQL23868	*SiAAAP12*	455	49,087.73	7.15	32.24	114.66	0.706	plas	–	11
KQL12891	*SiAAAP13*	464	50,964.29	8.14	33.2	96.55	0.431	plas	–	9
KQL13581	*SiAAAP14*	448	49,770.72	9.38	39.57	101.83	0.5	plas	–	9
KQL13582	*SiAAAP15*	448	49,262.31	9.28	39.53	105.62	0.584	plas	–	10
KQL14087	*SiAAAP16*	481	51,075	9.38	41.83	112.99	0.704	plas	–	11
KQL15415	*SiAAAP17*	494	53,509.62	8.45	30.34	102.85	0.553	plas	–	9
KQL16475	*SiAAAP18*	512	55,315.58	9.25	36.85	96.27	0.431	plas	–	11
KQL16487	*SiAAAP19*	584	62,588.7	5.67	51.76	102.5	0.376	plas	–	10
KQL10023	*SiAAAP20*	399	43,335.64	9.24	30.18	125.31	0.868	plas	–	11
KQL10735	*SiAAAP21*	582	63,487.07	8.08	57.36	101.48	0.158	plas	–	8
KQL11007	*SiAAAP22*	516	56,278.88	8.66	37.76	89.63	0.274	plas	–	10
KQL11008	*SiAAAP23*	408	44,684.85	6.58	34.81	99.46	0.575	plas	–	8
KQL11009	*SiAAAP24*	462	50,145.41	8.71	35.35	97.75	0.613	plas	–	10
KQL11321	*SiAAAP25*	459	49,906.73	6.2	27.55	125.34	0.731	plas	–	10
KQL04691	*SiAAAP26*	436	45,394.6	7.96	33.1	121.1	0.878	plas	–	11
KQL05737	*SiAAAP27*	436	45,589.27	8.04	36.52	117.64	0.664	plas	–	10
KQL05925	*SiAAAP28*	461	49,301.15	8.88	37.65	115.38	0.748	plas	–	10
KQL07529	*SiAAAP29*	460	47,991.38	9.21	37.29	114.83	0.753	plas	–	11
KQL07801	*SiAAAP30*	490	54,623.93	8.49	32.35	98.96	0.482	plas	–	10
KQL07809	*SiAAAP31*	460	49,149.77	8.91	29.78	111.35	0.692	plas	–	10
KQL07969	*SiAAAP32*	468	49,948.22	9.04	30.66	101.97	0.53	plas	–	10
KQL07970	*SiAAAP33*	423	45,451.23	9.02	39.55	104.68	0.614	plas	–	9
KQL00973	*SiAAAP34*	430	48,409.92	8.88	38.17	99.12	0.384	plas	–	9
KQL00974	*SiAAAP35*	466	52,689.09	9.22	42.67	101.85	0.385	plas	–	10
KQL00975	*SiAAAP36*	445	49,762.68	9.15	37.37	103.78	0.484	plas	–	11
KQL00976	*SiAAAP37*	446	49,761.85	9.06	31.73	100.99	0.491	plas	–	10
KQL00978	*SiAAAP38*	446	49,825.94	9.03	36.59	103.39	0.479	plas	–	11
KQL01185	*SiAAAP39*	446	49,606.56	9.19	38.44	103.16	0.481	plas	–	10
KQL01338	*SiAAAP40*	450	48,383.75	9.38	34.05	104.07	0.612	plas	–	9
KQL02035	*SiAAAP41*	445	47,209.15	9.57	38.86	96.94	0.554	plas	–	9
KQK96551	*SiAAAP42*	543	58,575.6	6.31	50.16	103.39	0.296	plas	–	9
KQK96813	*SiAAAP43*	469	49,596.39	9.42	39	119.23	0.724	plas	–	10
KQK96814	*SiAAAP44*	452	47,915.42	9.66	43.52	114.2	0.654	plas	–	10
KQK96923	*SiAAAP45*	415	44,083.37	9.52	29.47	114.94	0.774	plas	–	11
KQK97571	*SiAAAP46*	438	45,709.13	8.54	33.2	118.26	0.825	vacu	–	9
KQK97572	*SiAAAP47*	457	47,538.01	8.56	33.5	118.07	0.74	plas	–	10
KQK97590	*SiAAAP48*	434	47,117.43	8.97	38.76	103.82	0.627	plas	–	7
KQK97667	*SiAAAP49*	459	50,741.99	8.4	34.26	93.31	0.412	plas	–	9
KQK98458	*SiAAAP50*	525	56,703.08	9.61	48.48	105.37	0.506	plas	–	11
KQK99377	*SiAAAP51*	470	51,104.66	8.46	41.31	104.17	0.537	plas	–	8
KQK99460	*SiAAAP52*	480	52,147.41	7.02	34.24	96.96	0.465	plas	–	9
KQK99462	*SiAAAP53*	477	51,501.88	8.72	34.37	97.19	0.49	plas	–	9
KQK99463	*SiAAAP54*	477	51,502.73	8.27	32.99	96.54	0.504	plas	–	9
KQK99464	*SiAAAP55*	481	51,917.18	6.98	34.88	96.57	0.497	vacu	–	9
KQK99481	*SiAAAP56*	452	47,524.59	9.1	31.76	102.39	0.735	vacu	–	7
KQK93750	*SiAAAP57*	482	53,350.06	8.83	30.86	94.92	0.446	plas	–	10
KQK93944	*SiAAAP58*	473	50,880.06	8.61	38.09	97.99	0.502	plas	–	10
KQK93947	*SiAAAP59*	473	50,675.98	8.64	35.16	99.28	0.581	plas	–	9
KQK94422	*SiAAAP60*	482	50,810.81	9.23	38.24	113.51	0.639	plas	–	10
KQK89188	*SiAAAP61*	442	47,880.41	9.22	38.87	93.91	0.405	plas	–	9
KQK89520	*SiAAAP62*	541	59,701.37	9.1	40.43	92.53	0.385	plas	–	10
KQK90038	*SiAAAP63*	255	27,237.34	9.26	43.01	121.65	0.827	vacu	–	5
KQK91710	*SiAAAP64*	519	57,729.28	8.9	36.73	95.49	0.379	plas	–	10
KQK85918	*SiAAAP65*	466	51,178.73	8.55	30.18	106.18	0.466	plas	–	11

### Phylogenetic tree analysis of *SiAAAP* family

**Figure 1:**
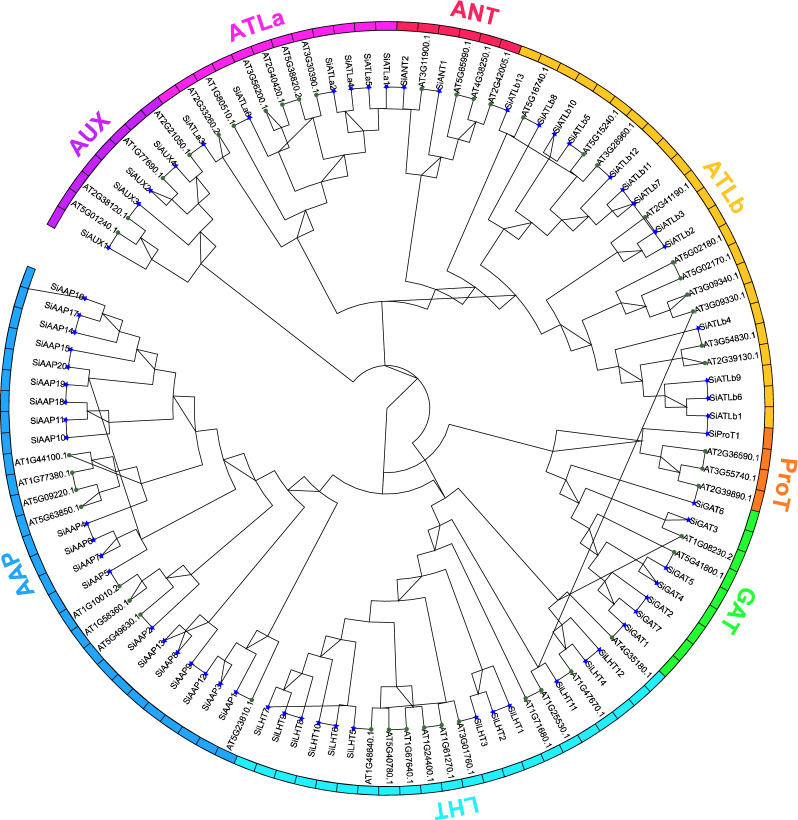
Phylogenetic tree of *AAAP* gene family in foxtail millet and *Arabidopsis.* A total of 47 AtAAAPs (represented by circles) and 65 SiAAAPs (represented by asterisk) were aligned. The phylogenetic tree was constructed in MEGA11.0. Different branch colors represent different subfamilies.

To analyze the evolutionary pattern of the *AAAP* family in foxtail millet, 65 SiAAAPs identified in this study and 47 AtAAAPs were used to construct a phylogenetic tree (Fig. 1). The results showed that the same subfamily in different species was clustered together, and SiAAAPs could be divided into eight subfamilies with the same classification as *Arabidopsis*, namely AAP, ATLb, LHT, GAT, ATLa, AUX, ANT, and ProT subfamilies. The largest subfamily was the *AAP* subfamily (20 members), and the second largest subfamily was ATLb (13 members). All ATLb members were distributed among 13 small clades under a large clade, suggesting internal variation among ATLb members. There were 12, 7, 6, 4, 2, and 1 member in the remaining six subfamilies, respectively.

### Gene structure, conserved motif, and conserved domain analysis of *SiAAAPs*

**Figure 2:**
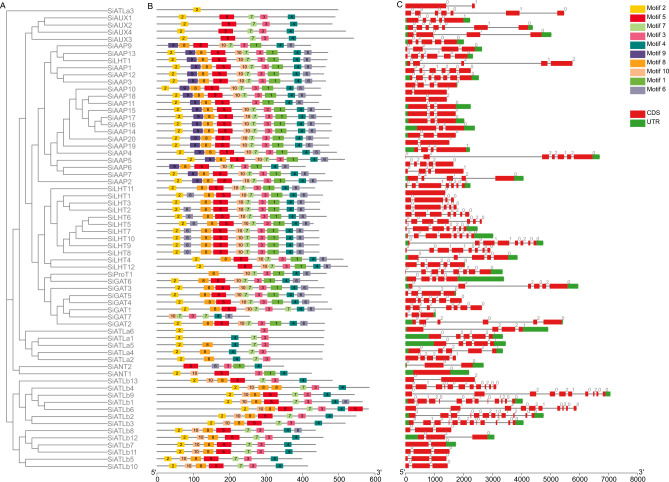
Phylogenetic relationship, conserved motifs, and gene structures of *SiAAAP* genes. (**A**) Phylogenetic tree of 65 *SiAAAP* genes. (**B**) Conserved motifs in the 65 SiAAAP proteins. Different colored boxes represent different conservative motifs. (**C**) Gene structures of *SiAAAP*s. CDS and UTR are indicated by red and green boxes, while introns are represented by black lines. The rulers at the bottom represent length.

By using the GFF annotation file of foxtail millet genome and the coding DNA sequence (CDS) sequence information of *SiAAAP* gene family, the conserved domains, conserved motifs, and gene structures of 65 *SiAAAP*s were visualized based on the phylogenetic tree of SiAAAP protein sequences and predicted by TBtools software (Fig. 2). Ten conserved motifs of *SiAAAP*s were identified by MEME online tool, whose length ranged from 6 to 50 amino acids. Motif 2 was located in the N-terminal AAAP domain region, and motif 4 and motif 6 were located in the C-terminal AAAP domain region.

Figure 2B shows that the position and number of conserved motifs varied in different subfamilies of foxtail millet. Except for *SiATLa1*, *SiATLa3*, and *SiATLa6*, which had small numbers of conserved motifs, the remaining members all contained at least five motifs. Except that *SiAAP6*, *SiAAP9*, and *SiGAT7* had no motif 2 and *SiATLa3* only had motif 2, the remaining members possessed motif 2, motif 3 and motif 4, indicating that motif 2, motif 3, and motif 4 are widely distributed and highly conserved in SiAAAPs. The arrangement and quantity of conserved motifs of most *SiAAAP*s were highly similar in the same subfamily, reflecting the evolutionary conservation of these genes and also indicating that the functions of these proteins are relatively conserved. However, some motifs are specific to some subfamilies, and motif 9 is specific to the SiAAP subfamily, indicating that *SiAAAPs* in different subfamilies have different functions.

We further analyzed the CDS information and untranslated regions (UTRs) of *SiAAAP*s. As shown in Fig. 2C, *SiAAAP* gene structure analysis showed that all *SiAAAP*s were broken genes except for *SiLHT1*. A total of 27 members showed both 5'and 3' UTRs; 14 members showed no UTR; and the remaining 14 members had 5'or 3' UTR. Most members in the same subfamily had the same or similar gene structure, but there were great variations in the number of exons among individual members. For example, among the 13 members of the ATLb subfamily, the largest number of exons was 13, and the smallest number of exons was 3; among the 12 members of the LHT subfamily, *SiLHT1* had only one exon, and *SiLHT8* had the largest number of exons (8). However, members of different subfamilies showed diversity in the number and position of exons and introns, with LHT, GAT, and ATLb having more abundant introns. Intronic phase is a conserved feature of eukaryotic gene structure and is associated with the evolution of introns in splicing. In terms of conservatism, phase 0 was the highest, phase 2 was the lowest, and phase 1 was in the middle. Combined with the phylogenetic tree, the intron number and phase analysis of each family member showed that the number of introns and exons was almost consistent among different members in the same subfamily, and most introns were cut in phase 0 mode. Gene structure analysis revealed that *SiAAAP*s are relatively diverse in gene structure, which may represent an evolutionary trend of gene functional diversification. Moreover, the position of exons and introns in the same subfamily is relatively conserved, indicating that members of the same subfamily have a close evolutionary relationship.

In summary, these results showed that there are some differences in amino acid conservation and conserved motifs among different subfamilies, suggesting that genes in different subfamilies may have different functions, and family members with the same or similar amino acids and conserved motifs may possess similar functions.

### Chromosome localization and collinearity analysis of *SiAAAPs*

**Figure 3:**
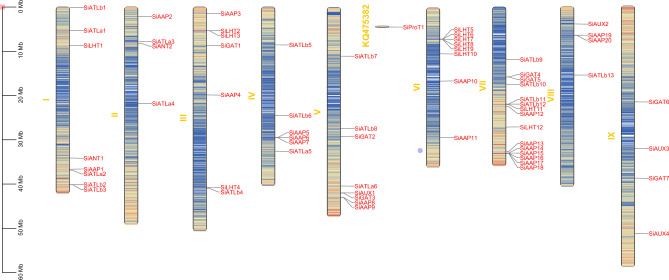
Chromosomal locations of *SiAAAP* genes. Genes are named based on their chromosomal locations. The number on the left side of the chromosome represents the chromosome number.

**Figure 4:**
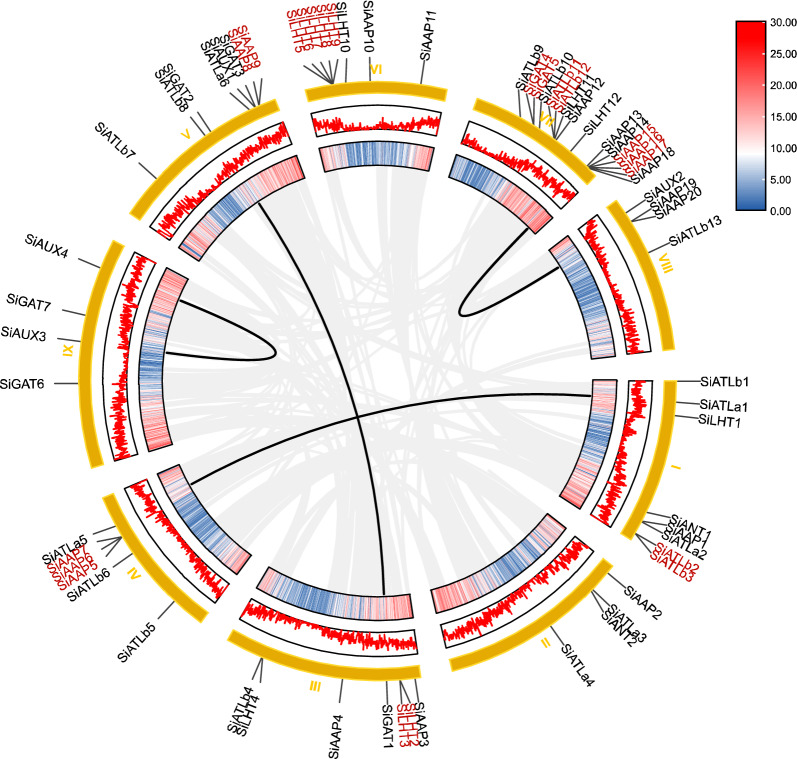
Inter-chromosomal collinear blocks of 65 *SiAAAP* genes. The circles arranged from outer to inner represent the chromosomes, gene density, and collinear blocks. Both linear and volcanic maps represent gene density. The gene names highlighted in red represent tandem repeats, and the gene pairs linked by black lines represent fragment repeats.

The MCScanX tool was used for mapping to perform chromosomal localization of 65 *SiAAAP*s (Fig. 3). Except that *SiProT1* was not located on any chromosome, the remaining 64 *SiAAAP*s were unevenly distributed on nine chromosomes, most of which were located at the proximal or distal end of chromosomes. Chromosome 7 had the most *SiAAAP*s (15). According to the determined criteria for tandem duplication genes, multiple collinear scanning kit and default parameters were used to analyze gene replication (E < E^−5^). If two homologous genes were separated by five or fewer genes, they were regarded as tandem duplication genes; if they were separated by more than five genes or distributed on different chromosomes, they were considered as segmental duplication. We identified 21 tandem duplication genes, accounting for 32% of the total, with eight gene clusters distributed on six different chromosomes, including three gene clusters in the AAP subfamily, two gene clusters in the LHT and ATLb subfamilies, and one gene cluster in the GAT subfamily. In addition, we identified four pairs of homologous genes involved in segmental duplication on the same or different chromosomes, accounting for 6% of the total (Fig. 4), including *SiGAT1* and *SiGAT2*, *SiATLa1* and *SiATLa5*, *SiAAP14* and *SiAAP19*, *SiAUX3* and *SiAUX4*. It can be inferred that these four pairs of genes may be formed due to large segmental duplication of chromosomes, and the amplification mode of the *SiAAAP* gene family is mainly tandem duplication.

### Synteny analysis of AAAP genes in foxtail millet, Arabidopsis, tomato, sorghum, rice, and maize

**Figure 5:**
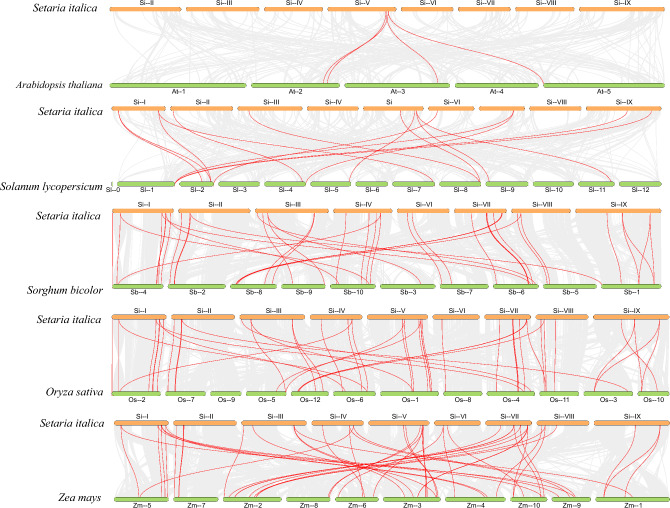
Synteny analysis of *AAAP* genes between *Setaria italica*, *Arabidopsis*, *Solanum lycopersicum*, *Sorghum bicolor*, *Oryza sativa*, and *Zea mays*. Gray lines represent the collinear relationship between foxtail millet and five other species and red lines represent collinear *AAAP* gene pairs.

To further investigate the phylogenetic mechanism of *AAAP* genes among different species, we analyzed the homologs between foxtail millet and five representative species, including two dicot plants (*Arabidopsis* and *Solanum lycopersicum*) and three monocot plants (*Oryza sativa*, *Zea mays*, and *Sorghum bicolor*) as shown in Fig. 5. Among these plant species, *Zea mays* had the most homologous genes (50 pairs), followed by *Oryza sativa* (48 pairs), *Sorghum bicolor* (42 pairs), *Solanum lycopersicum* (15 pairs), and *Arabidopsis* (4 pairs). In addition, *SiATLa1,2*, *SiAAP1*, *2*, *SiGAT1, SiLHT5,12*, *SiAUX3,4* shared homologous genes with five other plants except for *Arabidopsis*, suggesting that these genes may have existed before the divergence of monocotyledonous and dicotyledonous plants, and play important roles in the divergence of these plants. Overall, the *AAAP* genes of foxtail millet had the highest homology with those in *Zea mays*, and these highly homologous genes may have evolved from a common ancestor of different plants. In order to better understand the evolution process of the *SiAAAP* family, we estimated the time of duplication events of paralog pairs using the formula “T = Ks/2λ” (Table 2). The results showed that the Ka/Ks ratio of all gene pairs ranged from 0.1 to 0.46, and the predicted divergence time was 16.75–81.03 Mya (million years ago). The Ka/Ks ratio of duplicate gene pairs in foxtail millet was lower than 1. These results indicated that the evolution of the *SiAAAP* family was affected by strong purifying selection pressure, and gene functions tended to be conserved.

**Table 2:** Ka-Ks calculation for each pair of AAAP duplication genes in foxtail millet.

Duplicated gene pairs	Ks	Ka	Ka/Ks	Duplicated type	Time (Mya*)
*SiGAT4/SiGAT5*	0.38	0.16	0.43	Tandem duplication	29.30
*SiATLb2/SiATLb3*	0.75	0.20	0.27	Tandem duplication	58.07
*SiAAP8/SiAAP9*	1.05	0.34	0.33	Tandem duplication	81.03
*SiAAP15/SiAAP16*	0.22	0.03	0.15	Tandem duplication	16.75
*SiAAP16/SiAAP17*	0.22	0.02	0.11	Tandem duplication	17.03
*SiLHT8/SiLHT9*	0.70	0.07	0.10	Tandem duplication	54.05
*SiLHT5/SiLHT6*	0.36	0.08	0.22	Tandem duplication	27.36
*SiLHT6/SiLHT7*	0.51	0.13	0.25	Tandem duplication	39.02
*SiLHT7/SiLHT8*	0.69	0.12	0.17	Tandem duplication	53.20
*SiAAP8/SiAAP9*	1.05	0.34	0.33	Tandem duplication	81.03
*SiATLb11/SiATLb12*	0.56	0.26	0.46	Tandem duplication	42.99
*SiLHT2/SiLHT3*	0.25	0.10	0.39	Tandem duplication	19.09
*SiAAP5/SiAAP6*	0.73	0.23	0.31	Tandem duplication	55.98
*SiAAP6/SiAAP7*	0.34	0.12	0.34	Tandem duplication	25.90
*SiGAT1/SiGAT2*	0.53	0.12	0.22	Segmental duplication	40.38
*SiAAP14/SiAAP19*	0.46	0.15	0.33	Segmental duplication	35.11
*SiATLa1/SiATLa5*	0.94	0.10	0.10	Segmental duplication	72.6
*SiAUX3/SiAUX4*	0.44	0.10	0.23	Segmental duplication	33.48

### Analysis of cis-acting elements in *SiAAAP* promoters

**Figure 6:**
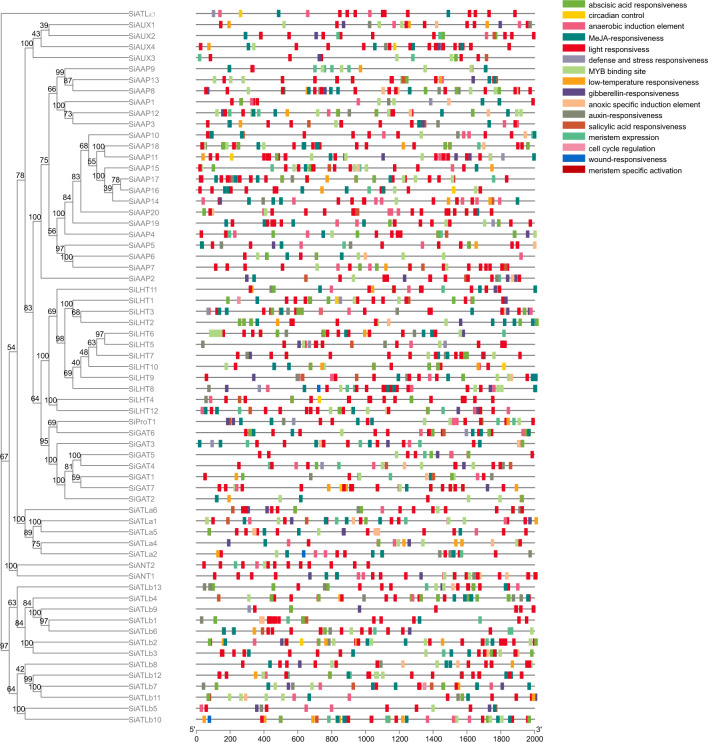
Cis-acting element analysis of *SiAAAP* promoter regions. Different colored boxes represent different types of cis-acting elements, while gray lines represent the length of gene promoters.

In order to investigate the regulatory role of *SiAAAPs*, we predicted and analyzed the cis-acting elements in the *SiAAAP* gene promoter region (Fig. 6). The cis-acting elements of *SiAAAP*s can be divided into four categories according to their functional annotations, including light-responsive elements, hormone-responsive elements (such as auxin, gibberellin, abscisic acid, salicylic acid, and jasmonic acid), stress-responsive elements (such as low temperature response elements, defense and stress response elements, damage response elements, antioxidant response elements, and hypoxia specific induction elements), and cis-acting elements related to growth and development (such as meristem expression or specific activation elements and MYB binding sites). Different *SiAAAP* members contained different types of cis-acting elements, but photoresponsive elements were present in the promoter region of all members, suggesting that the expression of *SiAAAP*s may be induced by light. A total of 35 *SiAAAP*s contained low temperature response elements; 15 contained defense and stress response elements; and 48 contained antioxidant response elements, indicating that *SiAAAP*s may play an important role in the process of abiotic stress response. Moreover, 64 *SiAAAP*s contained at least one hormone response element, suggesting that multiple hormones are involved in the regulation and expression of these genes, and there are certain synergistic or antagonistic relationships between different members. The most abundant stress response elements are enriched in the promoters of *SiATLa1* and *SiLHT1*, indicating that these two genes may play important regulatory roles in plant stress responses. Taken together, *SiAAAPs* may participate in various biological processes and play important roles in growth and stress response of foxtail millet.

### Enrichment analysis of *SiAAAPs*

**Figure 7:**
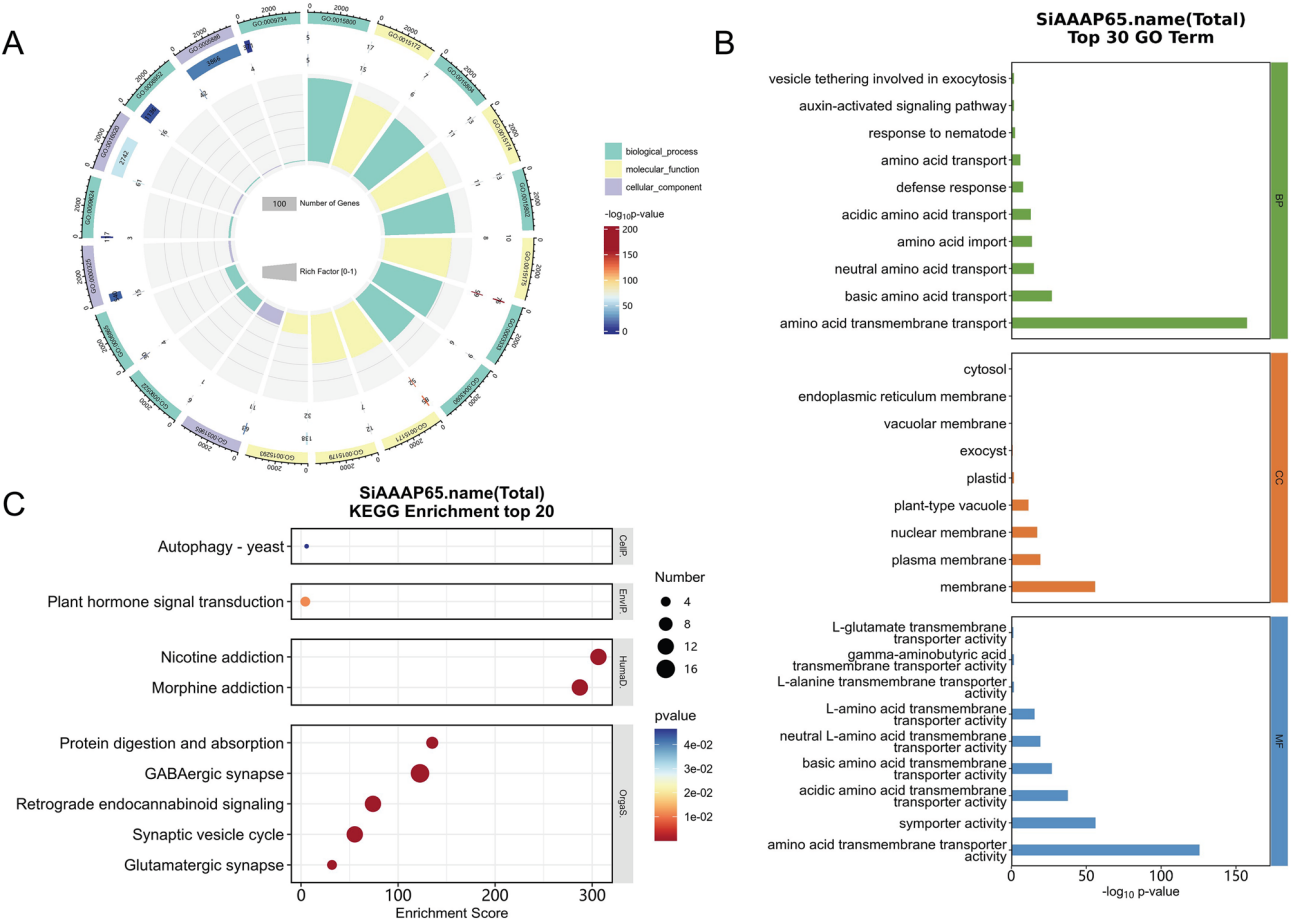
GO and KEGG enrichment analysis of *SiAAAP*s. (**A**) Circus plot of GO enrichment analysis of 65 *SiAAAP*s (all GO terms). (**B**) Histo plot of GO enrichment analysis of 65 *SiAAAP*s (top 30 enrichment terms). (**C**) Scatter plot of KEGG enrichment analysis of 65 *SiAAAP*s (top 20 enrichment terms and the picture is from www KEGG jp/KEGG/kegg1 html).

GO enrichment analysis of *SiAAAP*s showed (Fig. 7A) that these genes were enriched in 37 GO terms under three categories of biological process (19 GO terms), cell component (9 GO terms), and molecular function (9 GO terms). As shown in the Fig. 7B, in terms of biological process, *SiAAAP*s were mainly enriched in amino acid transmembrane transport, basic, neutral, and acidic amino acid transport, amino acid import, defense response, and auxin-activated signaling pathways. In terms of cell component, they were mainly enriched in membrane, plasma membrane, plant-type vacuole nuclear membrane, plastid, exocyst, and endoplasmic reticulum membrane. In terms of molecular function, they were mainly enriched in amino acid transmembrane transport activity, symporter activity, acidic amino acid transmembrane transporter activity, and other pathways. We found that the genes involved in the defense pathway of biological processes were concentrated in the LHT and AAP subfamilies, and the genes involved in the activation signaling pathway were concentrated in the AUX subfamily.

KEGG pathway enrichment analysis of *SiAAAP*s in Fig. 7C showed that these genes were significantly enriched in nine pathways, including GABAergic synapse, retrograde endocanna-binoid signaling, protein digestion and absorption, glutamatergic synapse and plant hormone signal transduction pathway.

### Expression analysis of *SiAAAPs* among different tissues

**Figure 8:**
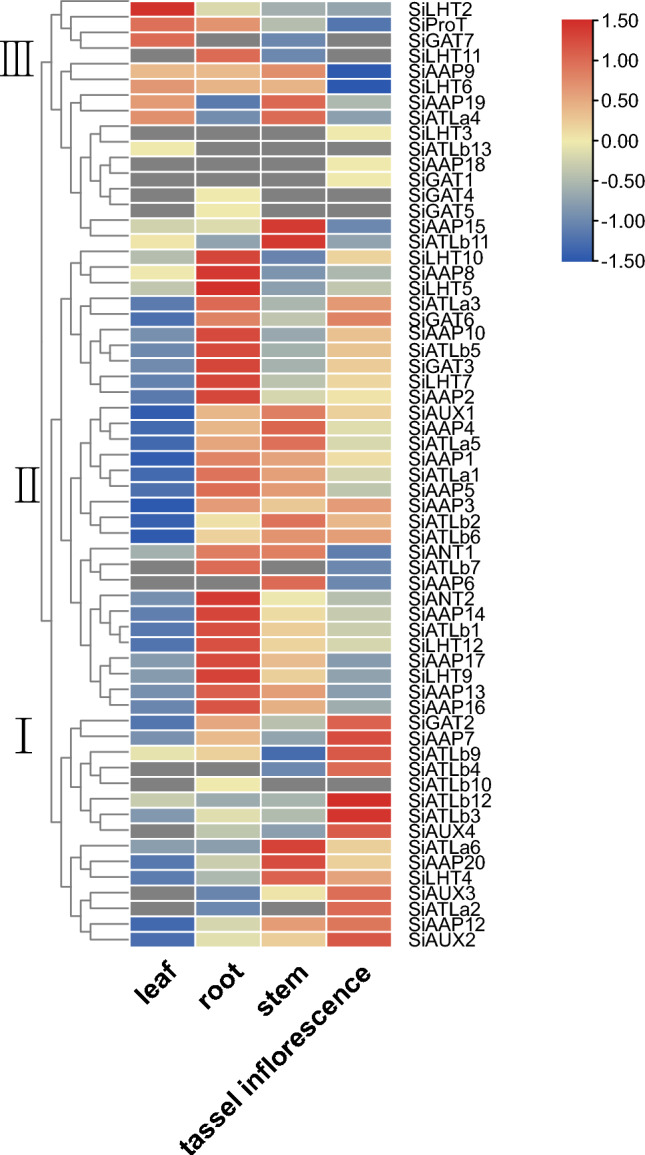
Tissue expression profile of the *SiAAAPs*. (A) Expression patterns of 65 *SiAAAP*s in the leaf, root, stem and tassel inflorescence tissues were examined. Red color represents up-regulated expression, blue color represents down-regulated expression. Based on the expression pattern, *SiAAAP*s were clustered into three major branches.

The expression pattern of genes is often associated with thier functions. *AAAP* genes have been found to have multiple functions in plant growth and stress resistance. Therefore, to further explore the expression pattern of *AAAP* genes, the RNA-seq data of foxtail millet variety 'xiaomi' in the database were used to map the expression profiles of *SiAAAP*s in roots, stems, leaves, and tassel inflorescence. Figure 8 shows that 61 of out of the 65 *SiAAAP*s were detected in at least one tissue and 46 *SiAAAP*s were detected in all tissues. According to the expression pattern, *SiAAAP*s were clustered into three branches. The first branch was highly expressed in the tassel inflorescence and lowly expressed in leaves and roots, indicating that it is important for flower formation. In the second branch, the expression of these genes was low in leaves, but high in roots, stems, and tassel inflorescence, indicating that these genes are particularly important for the whole growth and development of foxtail millet. Half genes in the third branch were highly expressed in leaves, and half of them were only expressed in specific tissues. For example, *SiGAT4* and *SiGAT5* were specifically expressed in roots; *SiATLb13* was specifically expressed in leaves; and *SiLHT3*, *SiGAT1,* and *SiAAP18* had specific expression in tassel inflorescence. By comparing the expression patterns of SiAAAP repeats, we found that *SiGAT4* and *SiGAT5* (tandem duplication genes) and *SiATLa1* and *SiATLa5* (segmental duplication genes) have similar expression patterns in different tissues, suggesting that they may have redundant functions. However, most of the overlapping genes showed different expression patterns, such as *SiA*TLb11 versus *SiATLb12*, *SiAAP6* versus *SiAAP7* (tandem duplication genes), and *SiAAP14* versus *SiAAP19* (segmental duplication genes).

### Prediction and correlation analysis of interacting proteins

**Figure 9:**
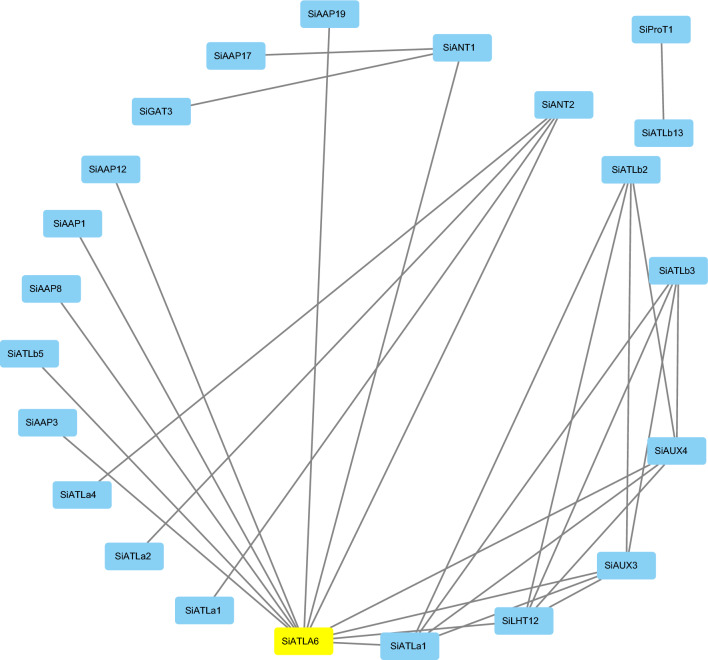
Prediction network of protein interactions for SiAAAPs**.** Each node represents a protein, and each edge represents an interaction. The protein with the most frequent interactions is highlighted with yellow.

Protein interaction networks^21^ can be used to predict functional orthologs within sequence homology clusters, which is of great significance for studying gene interactions and regulatory relationships. We therefore analyzed the interactions among individual SiAAAPs using the STRING online database, where each node represents a protein and the gray lines between two nodes indicate the interaction between proteins. Figure 9 shows that there were a large number of protein interactions in the whole regulatory network, as well as interactions between different subfamilies. There were 22 nodes and 30 edges between nodes, indicating that 22 proteins out of 65 SiAAAPs had co-expression. SiATLa6 interacted with 12 SiAAAPs, suggesting that it may be a core protein in the family, followed by SiAAP2, SiLHT12, SiAUX3, and SiAUX4, which could interact with four or five SiAAAPs, respectively, while the remaining SiAAAPs had few interactions. There were also interactions between different subfamilies of proteins, such as SiATLa6-SiAAP12, SiATLa6-SiAUX4, SiATLb13-SiProT1, SiANT2-SiATLa4, S Ilht12-SiATLb2 interactions. Therefore, it could be hypothesized that this family of proteins may be correlated when exerting their functions.

### Response of *SiAAAPs* to saline-alkali stress

**Figure 10:**
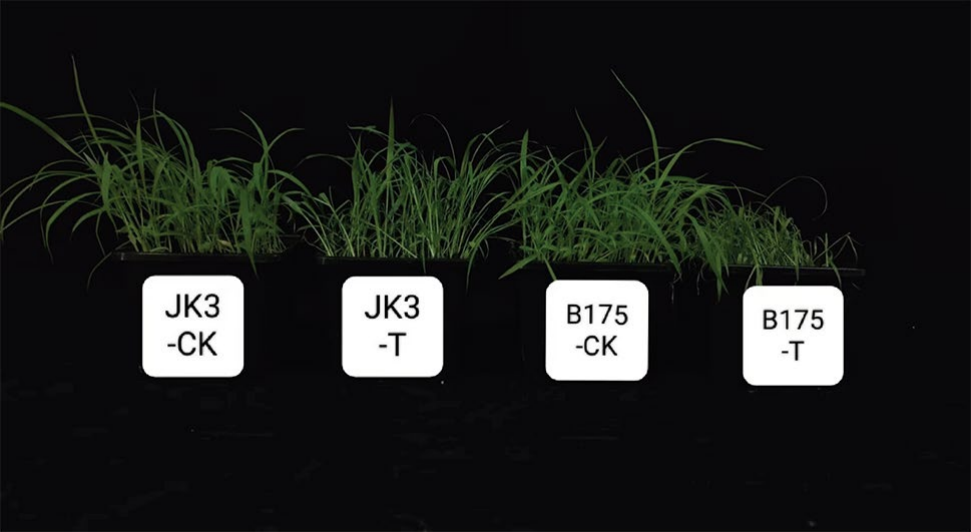
Phenotype observation of two millet varieties treated with salt and alkali stress for 48 h.

**Figure 11:**
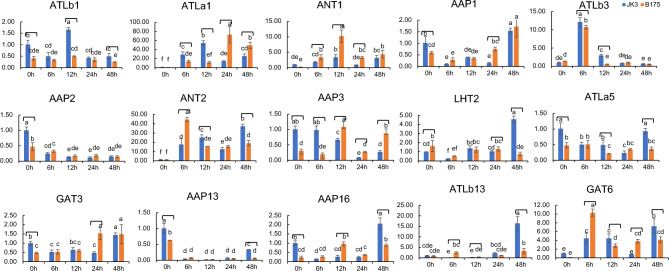
Expression patterns of the *SiAAAP* family under salt alkali stress treatment. The relative expression levels of 15 *SiAAAPs* were examined by qRT-PCR under salt alkali stress treatment for 0 h, 6 h, 12 h, 24 h, and 48 h. All samples were normalized with average expression of JK3 0 h. The standard error was represented by error bars, different letters indicate significant differences.

As predicted, multiple saline-alkali responsive elements were found in *SiAAAP* promoter regions (Fig. 6). In addition, previous studies have shown that *SiAAAPs* play a key role in response to abiotic stresses including saline-alkali stress. To verify the role of *SiAAAPs* under salt-alkali stress, we used qRT-PCR to investigate the expression levels of 15 randomly selected *SiAAAP*s (*SiATLa1, SiATLa5, SiATLb1, SiATLb3, SiATLb13, SiANT1, SiANT2, SiLHT2, SiGAT3, SiGAT6, SiAAP1, SiAAP2, SiAAP3, SiAAP13* and *SiAAP16*) from different subfamilies in leaves under salt-alkali treatment for 0, 6, 12, 24, and 48 h. At 48 h, the leaves of JK3 millet had little difference from the control, and the plants were straight and upright, while the B175 millet plants were wilted, as shown in Fig. 10. QRT-PCR results (Fig. 11) showed that multiple *SiAAAP*s were significantly differentially expressed in both tolerant variety JK3 and sensitive variety B175 at the same time compared with those in the control, and some members were only differentially expressed in B175. Among them, *SiGAT3* in B175 showed an upward trend in expression, while *SiAAP2* in B175 and JK3 showed a downward trend in expression, and *SiATLa1*, *SiANT1*, *SiATLb3*, SiANT2 in B175 and *SiATLb3* in JK3 showed a first increasing and then decreasing trend. *SiAAP1*, *SiAAP13* in B175 and *SiAAP1*, *SiATLa5*, *SiGAT3*, *SiAAP13*, *SiAAP16*, *SiATLb13* in JK3 showed a first decreasing and then increasing trend in expression. *SiATLb1*, *SiLHT2* in B175 and *SiATLb1* in JK3 showed a “descending, ascending, and then descending” trend in expression. *SiATLa5*, *SiAAP16*, *SiGAT6* in B175 and *SiATLa1*, *SiANT1*, *SiANT2*, *SiGAT6* in JK3 showed an “ascending, descending, and then ascending” trend in expression. *SiAAP3* in B175 and *SiLHT2* in JK3 showed a “descending, ascending, descending, and then ascending” trend of expression. These results indicated that all 15 genes showed differential expression under stress compared with the control, and showed different rhythms. In general, *SiATLb1, SiATLb3, SiANT1, SiANT2 SiAAP1, SiAAP2*, and *SiAAP13* showed the same expression trend in B175 and JK3, while *SiATLa1, SiATLa5, SiAAP3, SiAAP16, SiLHT2, SiATLb13 SiGAT3* and *SiGAT6* exhibited inconsistent expression patterns in B175 and JK3. The expression of *SiANT1* showed an “ascending, descending, ascending” trend in B175 with the extension of treatment time, and all reached the peak at 12 h, but consistently remained at low levels in JK3. The expression of *SiATLb1* in JK3 showed a “descending, ascending, descending” trend in expression, and reached the peak at 12 h. The expression of *SiATLb1* in B175 remained at a low level, and was down-regulated at 48 h compared with that at 0 h. These genes with inconsistent expression trends in the two varieties probably play a key role in the response to saline-alkali stress. Our results thus revealed a potential biological function of *SiAAAP*s in response to saline-alkali stress.

## Discussion

As a carrier of long-distance amino acid transport in plants, the *AAAP* gene family is closely related to plant development and stress response. At present, the genetic evolution patterns and biological functions of *AAAP* family members have been studied in many model plants, such as *Arabidopsis*^23^, *Alfalfa*^24^, *Moso bamboo*^25^ and *Pepper*^26^. However, the number and basic characteristics of *AAAP* family members in the C4 model plant foxtail millet have not been systematically characterized. In this study, 65 *SiAAAPs* were identified in foxtail millet. Compared with that in other plants, the number of family members is quite different, and is higher than that in *Arabidopsis* (49)^22^ and *Pepper* (53)^25^; however, it is similar to that in rice (71)^23^ and maize (71)^27^, which may be due to the fact that foxtail millet, rice, and maize belong to monocot grasses. The number of *AAAP* members in foxtail millet is larger than that in dicotyledonous plants, which promotes the expansion of *AAAP* family. In this study, the phylogenetic tree of foxtail millet and *Arabidopsis* showed that the eight subfamilies were highly consistent, indicating that the *AAAP* gene family is very old and became generally mature before the divergence of foxtail millet. In addition, we found that although the clades are similar to other *AAAP* gene families, there are great variations in the number of members in different subfamilies. This distribution pattern of subfamily numbers indicates species specificity, suggesting different degrees of expansion. For example, AAP subfamily members accounts for only 16.32% of the *AAAP* gene family in *Arabidopsis*, which is lower than that of LHT and ATLb subfamilies. In maize, AAP subfamily has the largest number of members, followed by LHT subfamily and ATLb subfamily, while GAT subfamily has the smallest number of members. Foxtail millet had the largest number of AAP members and smallest number of ProT subfamily members. This result may be related to the functional differences in *AAAP* gene family among different species. We further analyzed the gene structure, conserved motifs and TMs of SiAAAPs. We found that members of the same subfamily have a consistent conserved motif, exon/intron structure, but there are still structural differences in some genes, such as differences in exon number and the presence of ultra-long introns in some genes. There are more TMs in most SiAAAPs, and the number of TMs is reduced in individual genes, suggesting that most SiAAAPs are involved in the transport of amino acids with different specificities and properties, and mediate their transmembrane transport. This result indicates that the composition of TMs is closely related to gene function, and further indicates a direct connection between gene motifs and gene functions, and each subfamily is relatively conserved during evolution^26,28^.

As an important part of gene family expansion and functional diversity during gene evolution, gene duplication is also an important way for plants to cope with environmental changes during growth and development^29^, mainly through three pathways of chromosome segment duplication, tandem duplication and reverse transcription^30–32^. By analyzing the duplication of *SiAAAPs*, we found that there were 21 tandem duplication genes and only four pairs of segmental duplication genes, indicating that the evolution of *SiAAAPs* was mainly due to tandem duplication. Similar results have been reported in potato and *Arabidopsis*^33,34^. The amplification of *SiAAAP* family is more significant than that in *Arabidopsis* due to duplication events. For example, we detected 20 AAP subfamily members in foxtail millet, whereas only eight AAP members were identified in *Arabidopsis*. In addition, tandem duplication events mostly occur in the LHT subfamily and AAP subfamily, but less frequently in the ATLb and GAT subfamilies. The difference in gene quantity of different species and divergence of different subfamilies among species may be associated with the deletion and expansion of genes^35^. Gene replication often leads to changes in gene expression patterns, and the original functions of these genes may be preserved, sometimes leading to functional differentiation^36^. In this study, we found some duplication genes have similar expression patterns in different tissues, suggesting that they may have redundant functions. However, most of the overlapping genes showed different expression patterns. These results suggest that under purifying selection pressure, the expression or functional differentiation of repetitive genes helps plants adapt to diverse environments.

Gene expression patterns are often closely related to their biological functions, and vary among different plants^37^. Comprehensive analysis of gene expression levels can help infer gene functions during plant growth. In *Arabidopsis*, *AtAAAP1* is highly expressed in the seed cotyledon and endosperm, indicating that it is beneficial to the embryo mediated amino acid uptake process, and has a significant effect on seed yield^38,39^. Similarly, *RcAAP1* and *RcAAP2* in castor bean (*Ricinus communis*) are highly expressed in seeds, cotyledons and roots, but have relatively low expression in endosperm^40^. In this study, the *SiAAP* subfamily members were expressed in various tissues, indicating that they may be participated in different biological processes in different tissues of plants, which is the same as the previous research results^34,41–43^. Previous studies have shown that AUX subfamily genes in rice and potato are mainly expressed in roots^34,44^, and *AtAUX1* is also highly expressed in roots of *Arabidopsis*^45^. In this study, SiAUX subfamily genes had relatively high expression in both roots and tassel inflorescence, indicating that SiAUXs may not only be involved in root development but also play a role in inflorescence formation in foxtail millet. SiATLa6 and SiAUX4 are closely related in protein–protein interaction networks, suggesting that *SiATLa6* might also be involved in root formation. Previous studies have demonstrated that environmental stress can change the expression of defense-related genes in plants^46,47^, and large amounts of proline can be accumulated under a variety of environmental stresses, which was reported to be positively correlated with the ability of plants to resist stress in some studies^48–50^. Members of the ProTs subfamily have substrate specificity and mainly mediate the transport of compounds such as proline, betaine, and γ-aminobutyric acid^51^, and strongly induce the transmembrane transport of proline under abiotic stress to help plants resist stress^11,22,52^. In *Arabidopsis*, *AtProT2* is expressed in root epidermis and cortical cells, *AtProT3* is highly expressed in leaf epidermis^53^, and *SiProT1* has relatively high expression in roots and leaves in this study, indicating that SiProT1 may be induced to regulate the transmembrane transport of proline in plants treated with abiotic stress.

To further explore the relationship between the *SiAAAP* gene family and saline-alkali stress response, the expression of *SiAAAP*s under saline-alkali stress was analyzed by real-time fluorescent quantitative PCR. The results showed that the expression of *SiAAAPs* was induced by saline-alkali stress. In the two foxtail millet varieties, some genes such as *SiAAP16* and *SiGAT3* were rapidly induced and continuously expressed at the early stage of stress, while some genes *SiAAP2* and *SiAAP13* were inhibited at the early stage of stress. Moreover, GO enrichment analysis showed that the genes involved in defense stress response were all enriched in the AAP subfamily and the LHT subfamily, further indicating that AAP subfamily genes are resistant to saline-alkali stress. In *Arabidopsis*, *AtLHTs* are localized on the plasma membrane and involved in extensive amino acid transport. Some other studies have shown that Gln transported by *AtLHT1* can resist pathogens through the salicylic acid signaling pathway. In this study, *SiLHT2*, a member of the LHT subfamily, showed inconsistent expression patterns in JK3 and B175, indicating that LHT genes can participate in or regulate saline-alkali stress. ANT subfamily members are a unique family of sodium-dependent transporters^54^. In *Arabidopsis*, *AtANT1* can play a regulatory role in the transport of arginine, aromatic and neutral amino acids, especially in flowers and stems^55^. In this study, *SiANT1* and *SiANT2*, members of the ANT subfamily, were significantly up-regulated under saline-alkali stress in the two varieties, and *SiANT1* was more significantly up-regulated in B175 than in JK3, indicating the potential role of ANT genes in the saline-alkali stress tolerance in plants. In conclusion, the Si*AAAPs* may have potential in resistance to saline-alkali stress.

## Materials and methods

### Plant materials and saline-alkali stress treatments

In this study, salt-alkali tolerant millet variety JK3 and salt-alkali sensitive millet variety B175 were selected as experimental materials, and a pot experiment was conducted in an artificial climate chamber (JK3 millet were selected and provided by the Laboratory of coarse Grain Crops of Hebei Normal University of Science and Technology, and B175 millet was independently created and provided by Baoding Academy of Agricultural Sciences, Hebei Province, China). The conditions were set as day and night temperature 28 °C/22 °C, humidity 65%, and day and night duration of 12 h each. After being cultured to three leaves, 75% concentration artificial seawater was used for saline-alkali stress treatment, and the equal volume of distilled water was used as the control treatment. At 0, 6, 12, 24 and 48 h after treatment, the second and third leaves of foxtail millet were collected and sampled, immediately frozen in liquid nitrogen, stored in a  − 80 °C refrigerator for RNA extraction. Three biological replicates were used for each treatment.

### Identification of *AAAP* family in foxtail millet

We downloaded the entire foxtail millet genome data and annotation information files from Esembl Plant (http://plants.ensembl.org/index.html), and Arabidopsis genome data and annotation information file from TAIR (https://www.arabidopsis.org/). The protein sequences of the *AtAAAP* family were downloaded and used as query sequences. The TBtools software^56^ was used for blast comparison with the total protein sequence of foxtail millet, and the candidate genes of AAAP family in foxtail millet with E value < 1e^−5^ were screened. Moreover, the Hidden Markov Model (HMM) file of Aa_trans (PF01490) conserved domain of AAAP was downloaded from the Pfam database (http://pfam.xfam.org/)^57^. The Simple HMM Search command in TBtools software was used to search the total protein sequence of foxtail millet to screen candidate *SiAAAPs* genes of foxtail millet. The candidate genes identified by the two methods were combined and the duplicate genes were deleted to obtain the candidate genes of SiAAAP family. The candidate SiAAAP proteins which contain the Aa_trans were validated with SMART program (http://smart.embl-heidel‐berg.de/)^58^ and NCBI-CDD web server (https://www.ncbi.nlm.nih.gov/cdd/)^59^. The protein sequences containing functional domains in the analysis results of the two tools were intercrossed, and the sequences containing incomplete conserved domains were removed to determine the members of *SiAAAP* family.

### Analysis of physicochemical properties and subcellular localization of *SiAAAPs*

The Ex‐PASy (http://cn.expasy.org/tools) was used to analyze SiAAAP protein molecular weight, isoelectric point and feeling of water-borne and other physical and chemical characteristics^60^. SignaIP 4.1 Server (manuscript.v1-wang 20231019-2.docx) was employed to analyze SiAAAP protein signal peptide. TMHMM (http://www.cbs.etu.dk//cgi-bin/) was used to analyze the SiAAAP proteins across the membrane area. The WoLF PSORT (https://wolfpsort.hgc.jp/) was used for subcellular localization prediction AAAP protein sequence^61^.

### Phylogenetic tree, gene structure, conserved motifs and conserved domains analysis of *SiAAAPs*

MEGA11^62^ was used to align 65 SiAAAP protein sequences and 47 AtAAAP protein sequences. We built the phylogenetic tree using the neighbor-joining method with MEGA11, and the bootstrap value was set to 1000, and other parameters were set as default values. Modification of the evolutionary tree was done using the online tool ITOL (https://itol.embl.de/)^63^. They were named as SiAAAP1–SiAAAP65 according to their positions on chromosomes.

The exon and intron related information of *AAAP* family genes was obtained from the GFF file of foxtail millet genome annotation, so as to analyze the gene structure of *SiAAAP* family. The conserved motifs were analyzed using the online tool MEME (http://meme-suite.org/tools/meme)^64^, and the predicted value was set to 20. The domain and motif functions of *SiAAAP* were analyzed by Batch Web CD-search. The gene structure view function in TBtools was also used to visualize the distribution of *SiAAAP* gene structure, conserved domains, and conserved motifs.

### Prediction of promoter cis-acting elements

The upstream 2-kb sequence of the identified foxtail millet *AAAP* gene was submitted to PlantCARE (http://bioinformatics.psb.ugent.be/webtools/plantcare/html/)^65^. The website analyzed the cis-acting elements within the promoter region, such as hormone regulatory elements, growth and development related regulatory elements, or stress regulatory elements.

### Chromosome mapping and collinearity analysis and calculation of the Ka/Ks value

According to the annotation information of the foxtail millet genome, the chromosome location information of *SiAAAP* family was obtained, and the foxtail millet genome itself was compared with the multicollinearity scanning toolbox (MCScanX)^66^ to analyze the relationship between gene pairs for the tandem duplication and the segmental duplication. TBtools was used to map the chromosome location of *SiAAAP* genes and label tandem duplication genes, and the Basic Circos functional module was used to visualize the collinearity of *SiAAAP* genes. In order to further understand the evolution process of *SiAAAP* family, we selected the representative dicotyledonous species *Arabidopsis* and tomato (*Solanum lycopersicum*), and the representative monocotyledonous species rice (*Oryza sativa*), maize (*Zea mays*), and sorghum (*Sorghum bicolor*) to compare their genome sequences with that of foxtail millet, respectively, and obtained the collinear relationship between foxtail millet and other species. TBtools was used to draw the collinearity map between each species. DnaSP V5.0 was used to perform computational analysis of nonsynonymous (Ka) and synonymous substitution (Ks)^67^ and selection pressure analysis based on the value of Ka/Ks: 1) Ka/Ks > 1, replication event is a positive selection effect; 2) Ka/Ks < 1, gene replication event is purification selection effect, 3) Ka/Ks = 1, gene replication event is neutral selection effect^68^. The formula T = Ks/2λ × 10–6 Mya was used to calculate gene replication time, where T is gene replication time (unit: Million years, Mya), λ is the replacement rate (unit: synonymous site/year, λ = 6.56 × 10^–9^ in gramineae)^69^. All genome and annotation information were obtained from Esembl Plant database except for that of *Arabidopsis.*

### GO and KEGG enrichment analysis of *SiAAAPs*

The protein sequences and genome sequences downloaded from the genome database were sorted out, and the GO and KEGG background annotation files of mRNA were obtained. Based on gene ID and background annotation files, GO and KEGG enrichment analysis was performed, and gene function was annotated.

### Protein interaction network analysis of *SiAAAPs*

The identified SiAAAP protein sequences were submitted to a string database (https: //string-db.org/) and a functional protein network was constructed with a maximum number of five participants to predict the protein function of the *SiAAAP* genes.

### Expression patterns of *SiAAAPs* in various tissues

Using EMBL-EBI database (EMBL-EBI homepage|EMBL-EBI) of *SiAAAP* expression data of different organization in different periods, the data were submitted to TBtools software and analyzed, and the heat maps for *AAAP* family gene specific expression were drawn.

### Expression profile under saline-alkali stress and qRT-PCR analysis

Total RNA was extracted from foxtail millet leaves using the SteadyPure Plant RNA Extraction kit (purchased from Akoray Biological Co., LTD.), and its integrity was checked by 1% agarose gel electrophoresis, which showed that two bands of 18S and 28S were clearly visible. The Evo M-MLV reverse transcription premixed type kit was used for reverse transcription into cDNA. Using reverse-transcribed cDNA as template, primers (Table 3) were designed by Primer6 and synthesized by Shenggong Biological (Shanghai) Engineering Co., LTD. Real-time fluorescence quantitative PCR assay was performed using SYBR GreenI fluorescence quantitative assay kit. The reaction system included 0.3 μL of upstream and downstream primers, 0.75 μL of cDNA (obtained by 2:3 dilution of cDNA and ddH2O), 6.15 μL of ddH2O, 7.5 μL of qPCR SYBR Green Master Mix, and 15 μL of the total system. PCR reaction conditions were as 95 °C for 30 s; denaturation 5 s at 95 °C; 60 °C for 30 s; 40 cycles. Gene expression was calculated using 2^^−∆∆CT^ in triplicate for each sample using foxtail millet EF-1a as an internal control.

**Table 3:** qRT-PCR primer of *SiAAAP* gene family in foxtail millet.

Primer name	Forward primer(5’-3’)	Reverse primer(5’-3’)
*SiATLb1*	CGCAGAGCTACAGGCAATCCAT	GCAGGAGTGGCTTGACGAGATT
*SiATLa1*	TGACTCTGACCTTCGCATCCCA	GAGCAGCCCATCCAAGTTGAGC
*SiANT1*	GGAGGCGGCGAACAAGAAGAAG	GCCGAGGTTGGTGGTGATGATG
*SiAAP1*	CCCTCATCGCCGACTGCTACAT	CGAGCATCGTTGTTGCGTCCAT
*SiATLb3*	TTGCTGTTATCGGCTATCT	GCCAATGGGTTTATCACTA
*SiAAP2*	TCGGCGTCACCATCGGATACA	TCATGTTCGTCGTGTTGGAGGC
*SiANT2*	AACTGTGGTGGCGTCCTTCATC	CCGCCTCCACAAGGACATTGAC
*SiAAP3*	TCGCCGCCATCATGTCCTTCA	GCTTGAGCCACTCGCCAAACTT
*SiLHT2*	ACGATTGCGGAGATGACGACAG	ATGGCGGTGACATTGTGGAAGG
*SiATLa5*	TATGGGTGGCTGATGGGTGAGG	CCGGCTGATGTTGTTCCAGACA
*SiGAT3*	TGGTGCTGTGCCTCGCTTAC	CCGCCACCGTTGCCTGTATT
*SiAAP13*	ACACCATCACCGCAAGCACAAG	AGGCCGAACACCACCATGTAGG
*SiAAP16*	TGCACAACGGCACGAACCAC	CGATGACGGCGGTGATGATGTG
*SiATLb13*	CGCACTCCGTCATCAACATGGT	GCTCTCCGATGTCCTGGTAGGT
*SiGAT6*	ACCCTCGTCTCCGCAGCAAT	CTTCGCAGCATACCGCAATCCT
*SiEF-1a*	TGACTGTGCTGTCCTCATCA	GTTGCAGCAGCAAATCATCT

### Statement on guidelines

All experimental studies and experimental materials involved in this research are in full compliance with relevant institutional, national, and international guidelines and legislation .

## Conclusions

This study identified 65 *SiAAAPs* by genome-wide identification. We also comprehensively characterized the phylogenetic relationship, gene structure, conserved motifs, chromosomal location, gene duplication, promoter cis-acting elements, and saline-alkali stress-induced expression patterns. The results showed that the *SiAAAP* family could be divided into eight subfamilies, and chromosomal mapping showed that 65 *SiAAAP*s were unevenly distributed on nine chromosomes. Gene duplication analysis indicated that tandem duplication is the main force driving the expansion of *SiAAAP* family, and the family evolution is mainly affected by purifying selection pressure. Cis-acting element prediction revealed multiple stress response elements including saline-alkali stress response elements. The qRT-PCR results showed that the expression levels of *SiATLa1*, *SiAAP1*, and *SiGAT3* in both varieties increased significantly and continuously under saline-alkali stress, while those of *SiAAP3*, *SiLHT2*, and *SiAAP16* showed different trends between the two varieties. These results indicated that these genes are particularly critical in saline-alkali stress response. This study provides the complete *SiAAAP* gene family members, sequence structure characteristics, and expression pattern under saline-alkali stress, providing a reference for further studying the biological functions of each member of the *AAAP* family and the mechanism for saline-alkali tolerance in foxtail millet.

## Data Availability

All data generated or analysed during this study are included in this published article.
